# SARS-CoV-2 Seroprevalence among Healthcare, First Response, and Public Safety Personnel, Detroit Metropolitan Area, Michigan, USA, May–June 2020

**DOI:** 10.3201/eid2612.203764

**Published:** 2020-12

**Authors:** Lara J. Akinbami, Nga Vuong, Lyle R. Petersen, Samira Sami, Anita Patel, Susan L. Lukacs, Lisa Mackey, Lisa A. Grohskopf, Amy Shehu, Jenny Atas

**Affiliations:** Centers for Disease Control and Prevention, Hyattsville, Maryland, USA (L.J. Akinbami, S.L. Lukacs);; US Public Health Service, Rockville, Maryland, USA (L.J. Akinbami, S.L. Lukacs, L.A. Grohskopf);; Centers for Disease Control and Prevention, Fort Collins, Colorado, USA (N. Vuong, L.R. Petersen, L. Mackey);; Centers for Disease Control and Prevention, Atlanta, Georgia, USA (S. Sami, A. Patel, L.A. Grohskopf);; Epidemic Intelligence Service, Atlanta (S. Sami);; Region 2 South Healthcare Coalition, Detroit, Michigan, USA (A. Shehu, J. Atas)

**Keywords:** SARS-CoV-2, seroepidemiologic studies, hospitals, emergency responders, personal protective equipment, Detroit, public safety, 2019 novel coronavirus disease, severe acute respiratory syndrome coronavirus 2, zoonoses, coronavirus disease, COVID-19, viruses

## Abstract

To estimate seroprevalence of severe acute respiratory syndrome 2 (SARS-CoV-2) among healthcare, first response, and public safety personnel, antibody testing was conducted in emergency medical service agencies and 27 hospitals in the Detroit, Michigan, USA, metropolitan area during May–June 2020. Of 16,403 participants, 6.9% had SARS-CoV-2 antibodies. In adjusted analyses, seropositivity was associated with exposure to SARS-CoV-2–positive household members (adjusted odds ratio [aOR] 6.18, 95% CI 4.81–7.93) and working within 15 km of Detroit (aOR 5.60, 95% CI 3.98–7.89). Nurse assistants (aOR 1.88, 95% CI 1.24–2.83) and nurses (aOR 1.52, 95% CI 1.18–1.95) had higher likelihood of seropositivity than physicians. Working in a hospital emergency department increased the likelihood of seropositivity (aOR 1.16, 95% CI 1.002–1.35). Consistently using N95 respirators (aOR 0.83, 95% CI 0.72–0.95) and surgical facemasks (aOR 0.86, 95% CI 0.75–0.98) decreased the likelihood of seropositivity.

Healthcare, first response (e.g., firefighters, paramedics, emergency medical technicians), and public safety (e.g., law enforcement officers) personnel have served on the front lines of the coronavirus disease (COVID-19) pandemic response in several capacities. Many of these occupations require intensive interaction with persons with suspected or confirmed severe acute respiratory syndrome coronavirus 2 (SARS-CoV-2) infection. Both reverse transcription PCR (RT-PCR) testing for SARS-CoV-2 and assessing COVID-19 symptoms could be used to determine infection status, but not all infected persons develop symptoms or are tested within the necessary time window. Measuring antibodies to SARS-CoV-2 is necessary to inform our understanding of viral transmission dynamics in high-risk situations (*1*).

The Centers for Disease Control and Prevention (CDC) collaborated with the Michigan Department of Health and Human Services (MDHHS) Region 2 South and North Healthcare Coalitions to invite personnel working onsite in hospital, first response, and public safety settings to be tested for SARS-CoV-2 antibodies and to complete a web-based survey about workplace, occupation, use of personal protective equipment (PPE), and selected exposures. The primary study objective was to estimate the prevalence of SARS-CoV-2 antibodies among this population. A second objective was to describe associations between seroprevalence and participant and workplace characteristics.

## Methods

The MDHHS Region 2 South Healthcare Coalition area is the most populous region in Michigan and comprises Monroe, Washtenaw, and Wayne (including the city of Detroit) Counties. MDDHS Region 2 North Healthcare Coalition includes Macomb, Oakland, and St. Clair Counties. The Healthcare Coalitions coordinated with 27 hospitals and 7 MDHHS Medical Control Authorities (MCAs), which supervise and coordinate an emergency medical services (EMS) system for a geographic region, to invite employees to participate. The protocol was reviewed by CDC human subjects research officials, who determined the activity to be public health surveillance and exempt from full institutional review board review (*2*).

### Study Participants

Eligible participants for the serology survey included adults >18 years of age who worked onsite in a first response, hospital, or public safety setting and consented to phlebotomy and serum sample storage for confirmation of test results if needed. Persons were not eligible to participate if, in the 2 weeks before taking the survey, they reported having new symptoms of cough, shortness of breath, or change in sense of taste or smell, or had tested positive for SARS-CoV-2 by RT-PCR test using a nasal, throat, or saliva sample.

### Web-Based Survey

Participating agencies shared information about the secure web-based survey (Appendix Table 1) with employees using email and onsite marketing. There was no face-to-face recruitment. Participation was voluntary and individual results were not shared with employers. CDC did not have access to personal identifiers. The survey was drafted by the investigators, reviewed by CDC subject matter experts, and was designed to require <10 minutes to complete on a personal device. Upon survey completion, participants received information about blood collection sites at their workplace or a nearby MCA location.

### Specimen Collection and Testing

Blood samples were collected for SARS-CoV-2 antibody testing during May 18–June 13, 2020. Antibody testing was performed using the Ortho Clinical Diagnostics VITROS Immunodiagnostic Products Anti-SARS-CoV-2 IgG Test (https://www.orthoclinicaldiagnostics.com; specificity 100%, sensitivity 90%) (*3*). Results were reported to participants within 72 hours as negative (signal-to-cutoff ratio <1.0), positive (signal-to-cutoff ratio >1.0), or test not performed because of lipemia or insufficient volume.

### Statistical Analysis

Of 16,403 participants, 6 (0.04%) had samples that were unable to be tested and were excluded (n = 16,397). Percent SARS-CoV-2 antibody positivity and 95% CIs were calculated. Statistical testing was conducted using Cochran-Armitage trend tests for variables with ordinal categories (2-sided tests with α = 0.05). Non-Hispanic Native Hawaiian and other Pacific Islander (n = 31, 0.2%), Non-Hispanic American Indian/Alaska Native (n = 53, 0.3%), and other race (n = 320, 2.0%) participants were categorized as other race. The 398 (2.4%) participants who declined to report race were categorized separately and included in all analyses. 

Participants could choose multiple work locations; 17.5% chose >1 location. Each work location category was represented as a separate dichotomous variable (i.e., dummy variable) to enable modeling of non–mutually exclusive categories. Participants were provided with occupation categories and a free text option. The National Institute for Occupational Safety and Health (NIOSH) assisted with coding free-text responses using the NIOSH Industry and Occupation Computerized Coding System (*4*). No categories created from free-text options reached high sample size (n<100) and were coded as “other” except for technicians (e.g., dialysis, telemetry, surgery), which were combined into a “clinical technician” category (n = 365). 

Exposure to persons with confirmed COVID-19 (co-worker, household member, patient, and other person) was defined as contact within 6 feet for >10 minutes, but the question did not mention PPE use, which was assessed in separate questions. PPE use was dichotomized for each piece of equipment into “use all the time” (the recommended, or optimal, frequency when PPE is required) versus all other choices. Similarly, exposure to persons with confirmed COVID-19 was dichotomized into “yes” versus all other choices.

Differences between categories were assessed by nonoverlapping 95% CIs for percent positivity. Two participants were excluded from adjusted analyses (1 participant with missing housing information and 1 participant from a nonstudy hospital; n = 16,395). To account for clustering of participants by facility/agency, generalized estimating equations were used to model the likelihood of seropositivity. Covariates were chosen a priori to represent risk of exposure and infection. Model diagnostics performed with regression analysis did not show evidence of collinearity for work location (highest values for variance inflation factor = 1.4, and condition index = 4.1), which was represented by non–mutually exclusive dummy variables entered simultaneously into the multivariable model. No interaction terms were explored. SAS 9.4 software (https://www.sas.com) was used for all analyses. ArcGIS (ESRI, https://www.esri.com) was used to map seroprevalence by agency location.

## Results

**Figure 1:**
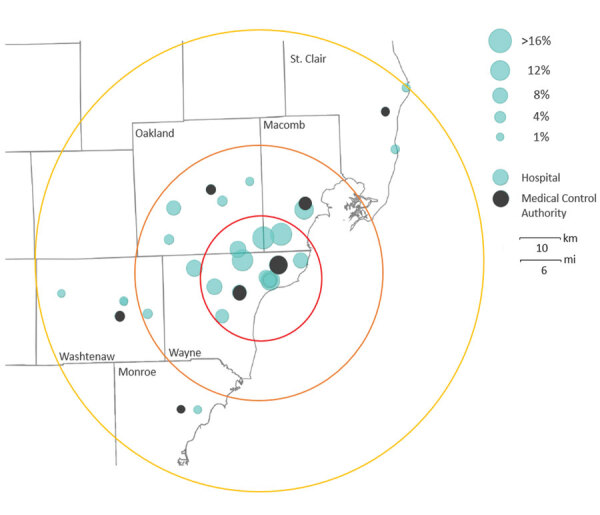
Seropositivity for SARS-CoV-2 among healthcare, first response, and public safety personnel, by hospital and Medical Control Authority agency location, Detroit metropolitan area, Michigan, USA, May–June 2020. Centroid: Detroit city center. Mean SARS-CoV-2 seroprevalence within 15 km was 11.0% (red), 15–30 km, 5.5% (orange), and 31–55 km, 1.8% (yellow). Base map source: ESRI ArcGIS map for Province of Ontario and Oakland County, Michigan (https://www.esri.com). SARS-CoV-2, severe acute respiratory syndrome coronavirus 2.

Of 16,397 participants, 6.9% (95% CI 6.5%–7.3%) were positive for SARS-CoV-2 IgG, indicating previous infection. In contrast, 2.7% (95% CI 2.5%–3.0%) reported having previously tested positive by RT-PCR using a nasal, throat, or saliva sample. Participant age ranged from 19 to 82 years (mean 42.1, SD 12.2). Seroprevalence was lower among those >65 years of age (3.2% of the sample; seroprevalence 3.5%, 95% CI 2.1%–5.4%) compared with all younger age groups (Table 1). Women, 68.6% of participants, had a similar seroprevalence to men. Non-Hispanic Black participants made up 7.3% of the sample and had the highest seroprevalence (16.3%, 95% CI 14.2%–18.5%) compared with other race/ethnic groups, including Non-Hispanic White participants (6.0%, 95% CI 5.6%–6.4%), who made up 78.4% of the sample. Seroprevalence among participants by facility ranged from 0.5% to 17.9% and was inversely related to distance of the facility from the Detroit geographic center. Seroprevalence was highest (11.0%, 95% CI 10.3%–11.7%) among participants at facilities within 15 km of Detroit’s center and lowest (1.8%, 95% CI 1.4%–2.2%) at locations 30–55 km away (Figure 1). Higher seroprevalence with closer proximity to Detroit was observed among most participants, regardless of occupation and healthcare setting (Appendix Figure 1). Among participants who reported close contact (within 6 feet) with a person with confirmed COVID-19 for >10 minutes, seroprevalence was highest among those with exposure to a household member (34.3%, 95% CI 30.2%–38.6%). Participants living in multiunit housing had higher seroprevalence compared with those living in single-family housing (8.4%, 95% CI 7.2%–9.8% vs. 6.7%, 95% CI 6.3%–7.1%).

**Table 1:** Seropositivity for SARS-CoV-2 among healthcare, first response, and public safety personnel, by demographic characteristics, Detroit metropolitan area, Michigan, USA, May–June 2020*

Characteristics	No. (%)	% Seropositive (95% CI)	
Total	16,397 (100.0)	6.9 (6.5–7.3)	
Age group, y†			
18–24	686 (4.2)	7.9 (6.0–10.2)	
25–34	4,885 (29.8)	6.9 (6.2–7.6)	
35–44	3,977 (24.3)	7.0 (6.2–7.9)	
45–59	5,222 (31.9)	6.9 (6.2–7.6)	
60–64	1,106 (6.8)	7.5 (6.0–9.2)	
>65	521 (3.2)	3.5 (2.1–5.4)	
Sex			
M	5,146 (31.4)	6.7 (6.0–10.2)	
F	11,251 (68.6)	7.0 (6.5–7.5)	
Race/ethnicity			
Non-Hispanic White	12,858 (78.4)	6.0 (5.6–6.4)	
Non-Hispanic Black	1,200 (7.3)	16.3 (14.2–18.5)	
Non-Hispanic Asian	1,097 (6.7)	7.3 (5.8–9.0)	
Hispanic	440 (2.7)	6.8 (4.7–9.6)	
Other‡	404 (2.5)	7.2 (4.9–10.2)	
Declined to answer	398 (2.4)	7.0 (4.7–10.0)	
Distance of work agency/facility from Detroit centroid			
<15 km	7,194 (43.9)	11.0 (10.3–11.7)	
15–30 km	4,677 (28.5)	5.5 (4.9–6.2)	
31–55 km	4,526 (27.6)	1.8 (1.4–2.2)	
Exposure to persons testing positive for COVID-19§			
Co-worker	6,799 (41.5)	10.0 (9.3–10.8)	
Household member	519 (3.2)	34.3 (30.2–38.6)	
Patient	10,389 (63.4)	7.8 (7.3–8.3)	
Other person	2,709 (16.5)	11.5 (10.3–12.7)	
Housing			
Multi-unit	1,762 (10.8)	8.4 (7.2–9.8)	
Single family	14,634 (89.3)	6.7 (6.3–7.11)	

*COVID-19, coronavirus disease; SARS-CoV-2, severe acute respiratory syndrome coronavirus 2. †Test for trend in seropositivity statistically significant at α = 0.05. ‡Other race/ethnicity includes non-Hispanic Native Hawaiian and other Pacific Islander, non-Hispanic American Indian and Alaska Native, and participants who indicated other race. §Categories are not mutually exclusive.

**Figure 2:**
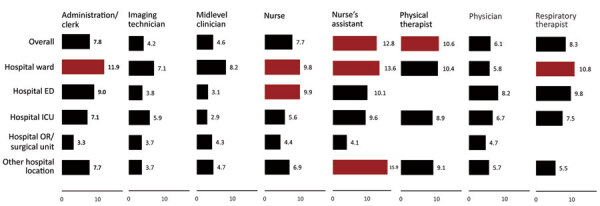
Seropositivity for SARS-CoV-2 among healthcare personnel by selected occupation and hospital work location, Detroit metropolitan area, Michigan, USA, May–June 2020. Red bars: lower 95% CI for percent positive is >6.9% (overall percent positive). Other hospital locations are all other locations not specifically listed in the chart (e.g., radiology, laboratory). Estimates not shown for categories with sample size <25 participants. ED, emergency department; ICU, intensive care unit; OR, operating room; SARS-CoV-2, severe acute respiratory syndrome coronavirus 2.

By work location, seroprevalence was highest among participants who worked in hospital wards (8.8%, 95% CI 8.0%–9.7%) and lowest among those working in police departments (3.9%, 95% CI 2.5%–5.8%) (Table 2). Within hospitals, lower seroprevalence was found among persons working in intensive care units (ICUs; 6.1%) and operating rooms or surgical units (4.5%) compared with participants working in wards (8.8%) and emergency departments (EDs; 8.1%). By occupation, the highest seroprevalence was found among nurse assistants (12.8%) and physical therapists (10.6%) and the lowest among laboratory technicians (3.4%) and police officers (4.0%). Seroprevalence also varied by hospital-based work locations and occupation (Figure 2). Among participants working on hospital wards, the lower bound of the 95% CI for seropositivity was higher than overall seroprevalence (6.9%) for nurse assistants (13.6%, 95% CI 10.2%–17.5%), administration/clerks (11.9%, 95% CI 7.0%–18.5%), respiratory therapists (10.8%, 95% CI 7.1%–15.6%), and nurses (9.8%, 95% CI 8.5%–11.2%). Nurse assistants who worked in an “other hospital location” (15.9%, 95% CI 8.2%–26.7%) and nurses who worked in EDs (9.9%, 95% CI 8.3%–11.7%) had similarly elevated seroprevalence.

**Table 2:** Seropositivity for SARS-CoV-2 among healthcare, first response, and public safety personnel, by work location, occupation, and PPE use, Detroit metropolitan area, Michigan, USA, May–June 2020*

Characteristic	No. (%)	% Seropositive (95% CI)	
Work location			
Hospital emergency department	3,614 (22.0)	8.1 (7.2–9.0)	
Hospital ward	4,766 (29.1)	8.8 (8.0–9.7)	
Hospital intensive care unit	3,973 (24.2)	6.1 (5.3–6.9)	
Hospital operating room/surgical	2,661 (16.2)	4.5 (3.7–5.3)	
Other hospital location	3,260 (19.9)	6.1 (5.3–7.0)	
Emergency medical services	550 (3.4)	5.3 (3.6–7.5)	
Fire services	1,008 (6.2)	5.0 (3.7–6.5)	
Police department	615 (3.8)	3.9 (2.5–5.8)	
Occupation			
Administration/clerk	964 (5.9)	8.0 (6.4–9.9)	
Clinical technician†	365 (2.2)	5.5 (3.4–8.3)	
EMT/medical first responder/paramedic‡	1,158 (7.1)	5.2 (4.0–6.6)	
Firefighter§	330 (2.0)	6.7 (4.2–9.9)	
Imaging technician	719 (4.4)	4.2 (2.8–5.9)	
Laboratory technician	293 (1.8)	3.4 (1.7– 6.2)	
Midlevel clinician	566 (3.5)	4.6 (3.0–6.7)	
Nurse	6,426 (39.2)	7.7 (7.1–8.4)	
Nurse assistant	641 (3.9)	12.8 (10.3–15.6)	
Other¶	688 (4.2)	6.8 (5.1–9.0)	
Other health#	200 (4.6)	7.5 (4.3–12.1)	
Pharmacist	321 (2.0)	4.4 (2.4–7.2)	
Physical therapist	235 (1.4)	10.6 (7.0–15.3)	
Physician	2,297 (14.0)	6.1 (5.1–7.1)	
Police/corrections officer	785 (4.8)	4.0 (2.7–5.6)	
Respiratory therapist	409 (2.5)	8.3 (5.8–11.4)	
PPE			
Gown use all the time	9,316 (56.8)	6.9 (6.4–7.5)	
Glove use all the time	11,887 (72.5)	7.0 (6.5–7.5)	
N95 respirator use all the time	7,316 (44.6)	6.9 (6.3–7.5)	
PAPR use all the time	695 (4.2)	7.6 (5.8–9.9)	
Goggles/face shield all the time	6,581 (40.1)	6.5 (5.9–7.1)	
Surgical facemask all the time	9,452 (57.6)	6.6 (6.1–7.1)	

*Work location categories are not mutually exclusive: 17.2% of participants reported >1 workplace. EMT, emergency medical technician; PAPR, powered air purifying respirator; PPE, personal protective equipment; SARS-CoV-2, severe acute respiratory syndrome coronavirus 2. †Includes dialysis, telemetry, cardiovascular, extracorporeal membrane oxygenation (ECMO), respiratory, emergency/critical care, anesthesia, endoscopy, orthopedic, surgical, neurodiagnostic, urology, audiology, and radiation technicians. ‡Includes firefighter/medical first responder. §Includes fire inspector and fire marshal. ¶Includes dietary staff, environmental staff, social worker/chaplain, maintenance staff, supervisor. #Includes dentist, medical examiner, orderly, phlebotomist, therapy aide, trainee.

Other occupational risk factors are included in the Appendix. Participants reported the average number of times per shift since March 1, 2020, in which they had participated in aerosol-generating procedures (Appendix Figure 2) and were given a list of examples for reference (Appendix Table 1). Seroprevalence generally increased with increasing procedure frequency (p = 0.04 by test for trend), with the highest percent positivity among those who participated in such procedures >25 times per shift on average (9.1%, 95% CI 7.4%–11.0%). Participants also reported how frequently they used each component of PPE, using a Likert scale. Overall, there was no pattern seen in percent antibody positivity with frequency of use with any PPE component (Appendix Table 2). Among those reporting ideal frequency of use (“all the time”) for a specific PPE component, seroprevalence was similar to the overall seroprevalence (Table 2).

**Figure 3:**
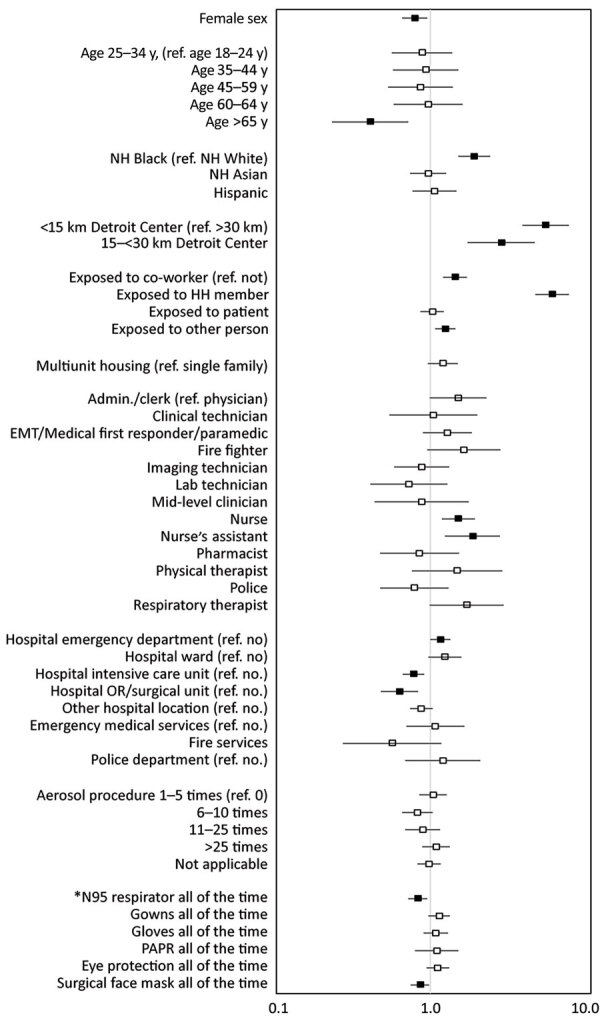
Adjusted odds ratios and 95% CIs for seropositivity for SARS-CoV-2 among healthcare, first response, and public safety personnel, Detroit metropolitan area, Michigan, USA, May–June 2020. Adjusted model was estimated using generalized estimating equations including all variables shown. Participants with other occupations, of other race/ethnicity, or who declined to provide their race/ethnicity are included in the models, but not shown as separate categories. Workplace variables are not mutually exclusive. Reference categories are noted in parentheses for each section. ED, emergency department; EMT, emergency medical technician; HH, household; Med 1st resp, medical first responder; NH, non-Hispanic; PAPR, powered air-purifying respirator; ref., reference; SARS-CoV-2, severe acute respiratory syndrome coronavirus 2. *Reference groups for personal protective equipment variables are all other responses with less frequency than “all the time.”

Multivariable adjustment using generalized estimating equations was performed (Figure 3; Appendix Table 3). Factors most strongly associated with likelihood of seropositivity were exposure to a household member with confirmed COVID-19 (adjusted odds ratio [aOR] 6.18, 95% CI 4.81–7.93) and working within 15 km of the Detroit center (aOR 5.60, 95% CI 3.98–7.89 compared with 30–55 km). Compared with physicians, occupations more likely to be seropositive included nurse assistant (aOR 1.88, 95% CI 1.24–2.83) and nurse (aOR 1.52, 95% CI 1.18–1.95). Working in a hospital ED was the sole location with increased adjusted odds of seropositivity (aOR 1.16, 95% CI 1.00–1.35). Consistently wearing an N95 respirator (aOR 0.83, 95% CI 0.72–0.95) or surgical facemask (vs. using them less than “all the time”) lowered the likelihood of being seropositive (aOR 0.86, 95% CI 0.75–0.98).

## Discussion

Healthcare, first response, and public safety personnel in 27 hospitals and 7 MCA areas were surveyed in the Detroit metropolitan area. Among these facilities, seroprevalence of SARS-CoV-2 IgG ranged from 0.5% to 17.9%, indicating wide variation in seroprevalence. A major role for community acquisition of SARS-CoV-2 infection is suggested by the strong association between seropositivity and working closer to the Detroit center and exposure to a household member with confirmed COVID-19. Workers remain vulnerable at home, where social distancing and PPE use may be difficult and likelihood of exposure during presymptomatic or asymptomatic periods is high. Similar patterns have been found in other studies of healthcare worker infections, in which community or household exposure to persons who tested positive for SARS-CoV-2 was the primary predictor of seroconversion (*5*,*6*). The geographic distribution of seroprevalence among first responders in this study was related to population-based cumulative case reporting in Michigan through April 30, 2020: higher percentage positivity for RT-PCR testing was reported for counties near the Detroit center (Oakland, Macomb, and Wayne [0.48%–1.04%]) versus lower levels for the outlying counties (Washtenaw, St. Clair, and Monroe [0.22%–0.32%]) (*7*).

Although 6.9% of participants were seropositive, only 2.7% reported a history of a positive RT-PCR test. This finding of higher seroprevalence compared with confirmed active infection is similar to other serology surveys of the general population (*8*–*10*). A study by Havers et al. estimated 6–24 times as many infections as the number of reported cases detected by RT-PCR (*8*). Our study revealed »2.5 times more infections than cases based on self-reported RT-PCR results. The 2.7% positivity for RT-PCR may be higher in the healthcare and first responder population compared with the general public (which ranged from 0.22% to 1.04% in the 6 Michigan counties) as a result of targeted and repeated testing of personnel in hospitals and emergency medical services settings (*11*). Even so, surveillance of these occupational groups in Detroit based on self-reported RT-PCR testing results would have identified a minority of infections.

Healthcare workers are known to be at occupational risk for SARS-CoV-2 exposure (*12*). Participants in occupations that may involve frequent and prolonged patient contact, such as nurse assistants and nurses (*13*,*14*), were more likely to be seropositive than physicians. Multivariable analysis revealed a weak association between lower seropositivity and consistent use of N95 respirators and surgical facemasks. Lower seroprevalence was observed among participants who reported high use of PPE despite shortages and reuse/extension protocols that could be hypothesized to lower the observed effectiveness of PPE. These and other confounding factors may obscure the role PPE plays in preventing infection, and it may be necessary to account for multiple factors in studies assessing the effect of PPE. The lower likelihood of seroprevalence associated with working in the controlled environments of a hospital ICU or surgical ward may reflect the impact of additional mitigation measures, including clear identification of infected persons and environmental and engineering controls (*15*). This pattern of lower seropositivity among staff in higher-risk versus lower-risk hospital settings has been described previously (*16*). However, even within healthcare work settings, some workers such as nurse assistants had a higher risk of infection than those in other roles. This finding highlights the concern that certain occupations may require additional focus on assessing and controlling factors related to transmission.

Together, these analyses of community and workplace factors show the contribution of community acquired infection to seropositivity among Detroit area healthcare workers. For 3 hospital settings (hospital ward, ED, and ICU) that could be compared across healthcare occupations, seropositivity rose with closer proximity of the facility to the Detroit center. This pattern suggests that regardless of occupation or work location, community acquisition was a common underlying factor of infection risk. There are 2 related implications. First, the observed impact of PPE may be reduced given the background impact of community acquisition of SARS-CoV-2 infection. Second, reducing community spread through population-based measures may directly protect healthcare workers on 2 fronts: reduced occupational exposure as a result of fewer infected patients in the less controlled workplace settings such as the ED, and reduced exposure in their homes and communities.

After adjusting for other factors, we found that women were less likely than men to be seropositive. This pattern was seen only in adjusted analysis; women’s lower risk may have been obscured by their disproportionate representation in the occupations at higher risk of infection. Women represented 69% of the sample but made up 86% of those in nursing and nurse assistance. 

Participants >65 years of age were less likely to be infected than younger workers. This pattern may be the result of measures to protect older workers from high-risk situations or from greater precautions taken among this group. A population study that also observed lower seroconversion among older persons found that older persons were less likely to live with a household contact (*17*). Seroconversion may also diminish with age in general (*17*), although other studies showed no pattern by age or higher seroprevalence among older persons (*18*,*19*). Participants of non-Hispanic Black race/ethnicity remained more likely to be seropositive than non-Hispanic white participants, even after adjustment. Community-level surveillance of COVID-19 infection and SARS-CoV-2 infection has demonstrated overrepresentation of minority groups in population-adjusted analyses (*20*,*21*). One hypothesis for the higher risk of infection among Black and Hispanic persons is employment in jobs without possibility of working remotely (*22*). Unfortunately, the survey did not collect information about occupation and workplace of household members. We speculate that the higher risk of exposure/infection among non-Hispanic Black versus non-Hispanic White participants in our study likely reflects uncontrolled confounding by factors for which data were not available.

Some limitations must be considered. The survey was a convenience sample with unknown representativeness: 80% of the 20,650 employees anticipated by MCA and hospital contacts to be eligible participated but agency participation varied, with highest participation among hospital personnel. The cross-sectional design precluded determining the source of exposure. In addition, comprehensive exposure data (e.g., travel, commuting, social exposures) were not collected. Because of the limited questionnaire length, PPE questions did not probe donning and doffing training, participant familiarity with PPE use, or reuse or extension protocols that may have affected effectiveness (*11*). No additional questions were asked about other workplace infection control practices. Another potential source of bias is the healthy worker effect, in which persons with prolonged COVID-19 infection or sequelae would not have been onsite to participate. Seroprevalence may be underestimated, given that the sensitivity of the antibody test was less than 100%. It is also possible that participants who were infected did not seroconvert (*23*; F. Gallais et al., unpub. data, https://www.medrxiv.org/content/10.1101/2020.06.21.20132449v1), but it is unknown whether lack of seroconversion may have occurred systematically between occupations (e.g., those exposed more intensely or with more severe illness may be more likely to develop antibodies) (*24*). Although more recent infections may have not been detected, it is unlikely that this varied systematically across groups. Strengths included coverage of a large number of personnel at hospitals and first response/public safety facilities and pairing antibody testing with questionnaire data to enable focus on a high-risk population.

Key implications for the risk of SARS-CoV-2 infection among healthcare, first response, and public safety personnel include the impact of community acquisition, increased odds of exposure associated with specific healthcare occupations, and the protection provided by PPE. Effects of interventions that could be further studied and implemented include providing alternative housing to healthcare workers during times or in areas of high community prevalence and ensuring that workers in high-risk occupations are given adequate PPE, specifically N95 respirators and surgical facemasks, as well as infection control training.

Appendix Additional information about SARS-CoV-2 seroprevalence among healthcare personnel in the Detroit metropolitan area.
