# Supplementary material for: SARS-CoV-2 Seroprevalence among Healthcare, First Response, and Public Safety Personnel, Detroit Metropolitan Area, Michigan, USA, May–June 2020
Source: Emerg Infect Dis. 2020 Dec;26(12):2863–71. doi: 10.3201/eid2612.203764 (PMC7706918; doi:10.3201/eid2612.203764)
Supplement: Appendix — Additional information about SARS-CoV-2 seroprevalence among healthcare personnel in the Detroit metropolitan area. [file 20-3764-Techapp-s1.pdf]

Article DOI: https://doi.org/10.3201/eid2612.203764

SARS-CoV-2 Seroprevalence among Healthcare, First Response, and Public Safety Personnel, Detroit Metropolitan Area, Michigan, USA, May–June 2020

Appendix

Appendix Table 1. Survey questionnaire administered to healthcare, first response, and public safety personnel, Detroit metropolitan area, May–June 2020

Question number	Questionnaire item	Response categories
1.	Name (not provided to CDC)	First<br>Last
2.	Home address (not provided to CDC)	Street<br>City<br>State<br>Zip
3.1	Phone number (mobile) (not provided to CDC)	Area code, phone number
3.2	Verify phone number (mobile) (not provided to CDC)	Area code, phone number
4.1	Email address (not provided to CDC)	(fill)
4.2	Verify email address (not provided to CDC)	(fill)
5.	County/borough of workplace	[scroll] (jurisdictions to provide list)<br>Other (fill)
6.	Date of birth (not provided to CDC)	MM/DD/YYYY
7.1	Sex at birth	Male<br>Female
7.2	Current gender (not provided to CDC)	Man<br>Woman<br>Transgender man/trans man/female-to-male (FTM)<br>Transgender woman/trans woman/male-to-female (MTF)<br>Genderqueer/gender nonconforming neither exclusively male nor female<br>Other (fill)<br>Decline to answer
8.	Sexual orientation (not provided to CDC)	Gay<br>Straight<br>Bisexual<br>Something else/not sure<br>Decline to answer
9.	Are you Hispanic or Latino/Latina?	Yes<br>No<br>Don’t know<br>Decline to answer
10.	What is your race?<br>(select all that apply)	White<br>Black/African American<br>Asian<br>American Indian or Alaska Native<br>Native Hawaiian or other Pacific Islander<br>Other<br>Decline to answer
10A	Please select your age group	18–29 years<br>30–39 years<br>40–49 years<br>50–59 years

Question number	Questionnaire item	Response categories
		60–64 years<br>65–69 years<br>70 years or older
11.1	What is your occupation?	Nurse (e.g., RN, APRN, LPN)<br>Midlevel clinician (e.g., PA, NP, nurse-midwife)<br>Nurse assistant (e.g., CNA)<br>Physician (e.g., MD, DO)<br>Student or trainee<br>Medical examiner<br>Mortuary technician<br>Police officer<br>Firefighter<br>Paramedic<br>Emergency medical technician<br>Medical first responder<br>Corrections officer or corrections staff<br>Respiratory therapist<br>Occupational/physical/speech therapist<br>Therapy aide/assistant<br>Pharmacist, pharmacist assistant<br>Diagnostic imagining technologist/technician<br>Clinical laboratory technologist/technician<br>Phlebotomist<br>Social worker/case manager<br>Administration, clerk<br>Medical records specialist/medical registrar<br>Dietary services staff<br>Environmental services staff<br>Maintenance staff<br>Orderly/transportation staff<br>Security guard
12.1	What is your main workplace or work setting?<br>(select all that apply)<br>(skip question 12.2 if non-hospital response)	Emergency department<br>Hospital ward/floor<br>Hospital ICU<br>Other hospital location<br>Fire station/department<br>Police station/department<br>Correctional facility<br>Medical examiner office<br>Other (fill)
12.2	What hospital do you work at?	[scroll] (jurisdictions to provide list)<br>Other (fill)
13.1	Since March 1st, how many weeks have you worked in this setting?	Weeks (scroll)<br>[min of 1, max of 20]
13.2	Since March 1st, on average, how many shifts did you work per week in this setting?	Shifts (fill)<br>[min of 1, max of 14]
13.3	Since March 1st, on average, how many hours did you work per shift?	Hours (scroll)<br>[min of 1, max of 24]
15.1	In the course of your work, how often did you use personal protective equipment (PPE) when within 6 feet of a person with suspected or confirmed COVID-19?<br>All the time<br>Most of the time<br>Sometimes<br>Rarely/never<br>Not applicable	Gown (Go to Q 18)
15.2		Gloves (Go to Q 18)
15.3		N95 respirator (Go to Q 16)
15.4		Powered air purifying respirator (PAPR) (Go to Q 17)
15.5		Goggles or face shield (Go to Q 18)
15.6		Surgical facemask (Go to Q 18)
15.7		Other respirator (Go to Q 18)
16.	In the last year, have you been fit tested for the respirator you wore?	Yes<br>No<br>Don’t know

Question number	Questionnaire item	Response categories
17.	In the last year, have you been trained to use the respirator you wore?	Yes<br>No<br>Don't know
18.	Since March 1, on average, how many times per shift did you participate in any aerosol-generating procedures for suspected or confirmed COVID-19 patient(s)?<br>Examples:<br>Open suctioning of airways<br>Sputum induction<br>Cardiopulmonary resuscitation<br>Endotracheal intubation and extubation<br>Noninvasive ventilation (e.g., BiPAP, CPAP)<br>Bronchoscopy<br>Manual ventilation	More than 25 times<br>10–25 times<br>6–10 times<br>1–5 times<br>0<br>Not applicable
19.1	Did you spend more than 10 minutes within 6 feet of a coworker who tested positive for COVID-19?	Yes<br>No<br>Don't know
19.2	Did you spend more than 10 minutes within 6 feet of a household member who tested positive for COVID-19?	Yes<br>No<br>Don't know
19.3	Did you spend more than 10 minutes within 6 feet of any other person who tested positive for COVID-19?	Yes<br>No<br>Don't know
20	How many times were you tested for COVID-19? (nasal, throat, or saliva sample)	(scroll)<br>[indicate 0 if none] (if 0 Go to 23.1)
21.	Have you ever had a positive result for COVID-19?	Yes<br>No<br>Don't know
22.	Approximately, when was the last time you were tested for COVID-19 in 2020 (excluding blood testing for exposure to SARS-CoV-2)?	MM/DD<br>Don't know
23.1	Since March 1, have you experienced any of the following symptoms?	Fever
		Chills
		Cough (new onset or worsening of chronic cough)
		Sore throat
		Shortness of breath or difficulty breathing
		Vomiting
		Diarrhea ($\geq$3 looser than normal stools/24hr period)
		Muscle aches
		New loss in sense of smell or taste
		Headache (new onset or worsening headache)
		Other (fill)
24.	When did these symptoms start? (estimate as best as possible)	MM/DD<br>Don't know
25.	Did you seek healthcare for these symptoms?	Yes<br>No<br>Don't know
26.	Were you hospitalized for COVID-19 illness?	Yes<br>No<br>Don't know
27.	Do you live in a single-family home or multi-unit housing (like an apartment)?	Single family<br>Multi-unit<br>Other (fill)
28.	Number of household members currently in the residence including yourself (resident, family, live-in staff, roommates, and long-term visitors)	Specify (scroll)
29.	What is your height?	Feet<br>Inches
30.	What is your weight?	Weight in pounds
31.1	Do you have any of the following chronic medical conditions?	Diabetes
		Hypertension (high blood pressure)
		Chronic heart disease

Question number	Questionnaire item	Response categories
		Chronic kidney disease
		Chronic liver disease
		Asthma
		COPD/emphysema/chronic bronchitis
		Immunosuppressive condition (e.g., HIV, autoimmune disease)
		Immune-weakening medication or therapy (e.g., cancer treatment)
32.	Since March 1, have you been or are you currently pregnant?<br>(only when sex at birth is female)	Yes<br>No<br>Don't know

Appendix Table 2. Percent seropositive for SARS-CoV-2 among healthcare, first response, and public safety personnel, by frequency of personal protective equipment (PPE) use, Detroit metropolitan area, May–June 2020

PPE	Use	No.	Percentage	Percent seropositive	p value*
Gowns	All the time	9,316	56.8	6.9	0.12
	Most of the time	3,335	20.3	7.6	
	Sometimes	1,285	7.8	6.0	
	Rarely or never	1,935	11.8	5.9	
	Not applicable	526	3.2	7.2	
Gloves	All the time	11,887	72.5	7.0	0.47
	Most of the time	2,150	13.1	6.7	
	Sometimes	1,171	7.1	6.8	
	Rarely or never	865	5.3	6.4	
	Not applicable	324	2.0	5.6	
N95 respirator	All the time	7,316	44.6	6.9	0.14
	Most of the time	3,537	21.6	7.8	
	Sometimes	2,252	13.7	6.8	
	Rarely or never	2,574	15.7	5.9	
	Not applicable	718	4.4	6.0	
Powered air-purifying respirator	All the time	695	4.2	7.6	0.77
	Most of the time	449	2.7	4.5	
	Sometimes	1,149	7.0	7.6	
	Rarely or never	11,560	70.5	6.7	
	Not applicable	2,544	15.5	7.5	
Goggles or face shield	All the time	6,581	40.1	6.5	0.43
	Most of the time	4,046	24.7	7.3	
	Sometimes	2,091	12.8	7.9	
	Rarely or never	2,919	17.8	6.5	
	Not applicable	760	4.6	6.8	
Surgical facemask	All the time	9,452	57.6	6.6	0.99
	Most of the time	2,901	17.7	8.1	
	Sometimes	1,756	10.7	8.3	
	Rarely or never	1,801	11.0	5.4	
	Not applicable	487	3.0	5.8	

*Cochran-Armitage trend test, omitting the "not applicable" category.

Appendix Table 3. Adjusted odds ratios and 95% confidence intervals for seropositivity for SARS-CoV2 among healthcare, first response, and public safety personnel, Detroit metropolitan area, May–June 2020*

N = 16,395	Odds ratio	95% LCI	95% UCI
Sex			
Men	ref		
Women	**0.79**	**0.65**	**0.95**
Age group, years			
18–24	ref		
25–34	0.89	0.56	1.39
35–44	0.93	0.57	1.52
45–59	0.86	0.53	1.40
60–64	0.97	0.58	1.62
≥65	**0.41**	**0.23**	**0.72**
Race/ethnicity			
Non-Hispanic white	ref		
Non-Hispanic Black	**1.92**	**1.52**	**2.43**
Non-Hispanic Asian	0.97	0.74	1.26
Hispanic	1.06	0.76	1.48
Other	0.95	0.67	1.35
Decline	0.98	0.69	1.41

N = 16,395	Odds ratio	95% LCI	95% UCI
Distance of workplace from Detroit center			
<15 km from Detroit center	**5.60**	**3.98**	**7.89**
15–30 km from Detroit center	**2.88**	**1.74**	**4.77**
31–55 km from Detroit center	ref		
Exposure to COVID-19 (ref = no or unknown exposure)			
Co-worker	**1.45**	**1.21**	**1.73**
Household member	**6.18**	**4.81**	**7.93**
Patient	1.03	0.87	1.22
Other person	**1.25**	**1.08**	**1.45**
Housing			
Single family	ref		
Multi-unit	1.20	0.96	1.50
Occupation			
Physician	ref		
Administration/clerk	1.51	0.99	2.31
Clinical technician	1.04	0.54	2.02
EMT/medical first responder/paramedic	1.29	0.90	1.85
Firefighter	1.65	0.95	2.84
Imaging technician	0.88	0.58	1.33
Lab technician	0.73	0.41	1.29
Midlevel clinician	0.88	0.44	1.77
Nurse	**1.52**	**1.18**	**1.95**
Nurse assistant	**1.88**	**1.24**	**2.83**
Other	1.19	0.84	1.70
Other health occupation	1.19	0.63	2.25
Pharmacist	0.85	0.47	1.53
Physical therapist	1.49	0.76	2.92
Police	0.79	0.47	1.31
Respiratory therapist	1.72	0.99	2.99
Workplace (ref = did not work in specified workplace)†			
Hospital emergency department	**1.16**	**1.00**	**1.35**
Hospital ward	1.24	0.97	1.58
Hospital intensive care unit	**0.78**	**0.66**	**0.91**
Hospital operating room/surgical unit	**0.63**	**0.48**	**0.83**
Other hospital location	0.87	0.74	1.03
Emergency medical services	1.08	0.70	1.66
Fire services	0.56	0.27	1.18
Police department	1.20	0.69	2.10
Aerosol-generating procedure frequency			
0 times	ref		
1–5 times	1.04	0.85	1.28
6–10 times	0.83	0.66	1.04
11–25 times	0.89	0.69	1.16
>25 times	1.09	0.89	1.34
Not applicable	0.98	0.83	1.17
Personal protective equipment use frequency			
N95 respirator all the time	**0.83**	**0.72**	**0.95**
Gowns all the time	1.14	0.97	1.34
Gloves all the time	1.09	0.90	1.30
PAPR all the time	1.10	0.80	1.52
Eye protection all the time	1.12	0.94	1.32
Surgical facemask all the time	**0.86**	**0.75**	**0.98**

*Adjusted model estimated using generalized estimating equations including all variables shown. Bolded estimates are statistically significant. EMT, emergency medical technician; LCI, lower confidence interval; PAPR, powered air-purifying respirator; UCI, upper confidence interval.
†Workplace not mutually exclusive.

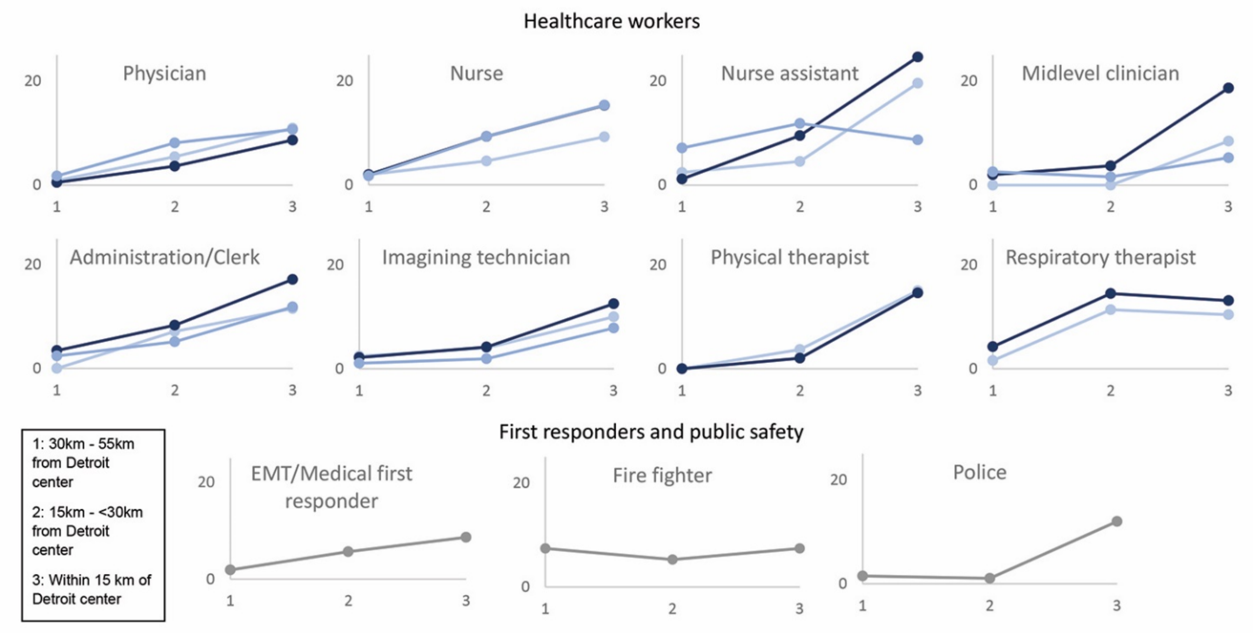

Appendix Figure 1. Percent seropositive by proximity to Detroit geographic center, by selected occupation and hospital workplace among healthcare personnel, and selected occupation among first response and public safety personnel, Detroit metropolitan area, May–June 2020.

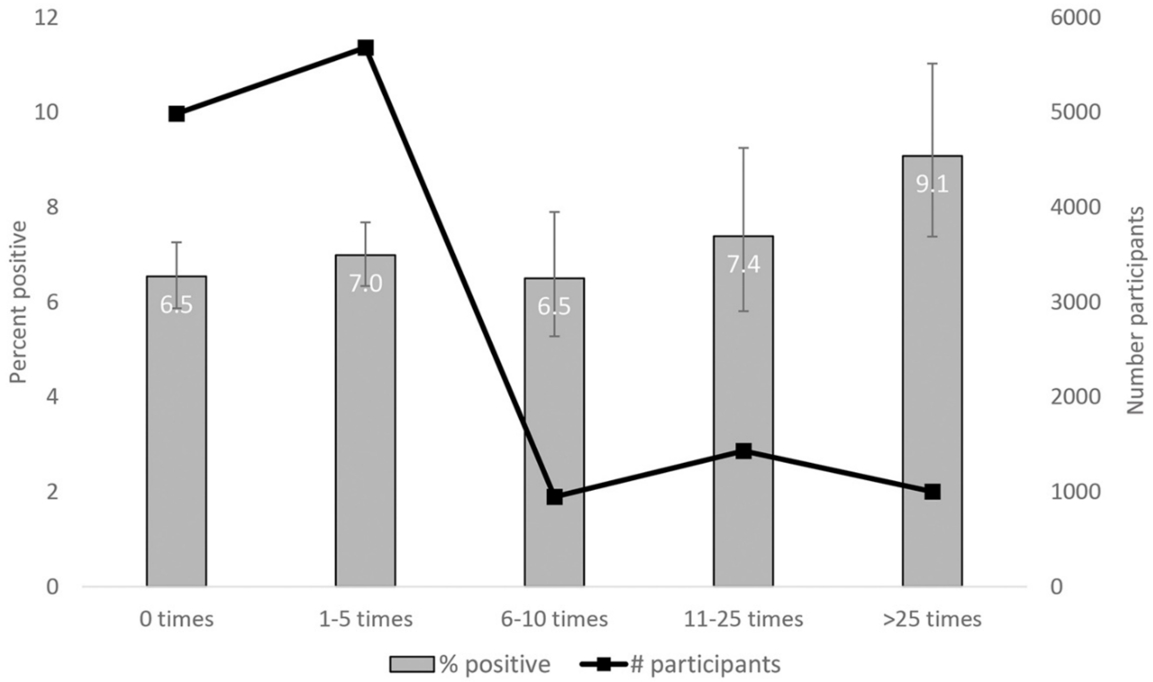

Appendix Figure 2. Percent seropositive with 95% confidence interval and number of participants by average number of aerosol-generating procedures per shift among healthcare, first response, and public safety personnel, Detroit metropolitan area, May–June 2020 (n = 14,047). Excludes participants who reported “not applicable” (n = 2,350). Cochran-Armitage test trend p = 0.037 for percent positive.
